# The COVID-19 alcohol paradox: British household purchases during 2020 compared with 2015-2019

**DOI:** 10.1371/journal.pone.0261609

**Published:** 2022-01-19

**Authors:** Peter Anderson, Amy O’Donnell, Eva Jané Llopis, Eileen Kaner

**Affiliations:** 1 Population Health Sciences Institute, Newcastle University, Newcastle upon Tyne, England; 2 Department of Health Promotion, Faculty of Health, Medicine and Life Sciences, Maastricht University, Maastricht, Netherlands; 3 Institute for Mental Health Policy Research, Centre for Addiction and Mental Health, Toronto, Ontario, Canada; 4 ESADE Business School, Ramon Llull University, Barcelona, Spain; University of Texas at Arlington, UNITED STATES

## Abstract

British supermarket-panel data suggest no increases in overall sales and purchases of alcohol following COVID-19 lockdowns, yet survey and mortality data suggest otherwise. This paper attempts to unravel the paradox. Based on purchase data of 79,417 British households from Kantar Worldpanel, we undertake controlled interrupted time series analysis of the impact of COVID-19 confinement introduced on 23^rd^ March 2020, and variably applied during 2020, compared to purchases during 2015 to 2019 as controls. We also undertook Poisson regression analyses to estimate if changes in purchases differed by household socio-demographic and economic factors. Excess off-trade household alcohol purchases (expressed as grams of ethanol) following the introduction of confinement, were 29.2% higher (95% CI = 25.8% to 32.5%) for the post-confinement months of 2020, being larger until mid-July 2020 (37.5%, 95%CI = 33.9 to 41.26%) when pubs re-opened with restrictions, and smaller (24.6%, 95%CI = 21.6 to 27.7) thereafter. During the time of complete pub closures, and fully adjusting for no on-trade purchases, household purchases of alcohol did not change when compared with the same time period during 2015–2019 (coefficient = -0.9%, 95%CI = -5.6 to 3.8). Excess purchases from 23^rd^ March to 31^st^ December 2020 varied by region of Great Britain, being higher in the north of England, and lower in Scotland and Wales. Excess purchases were greater in the most deprived households, compared with the least deprived households. Excess purchases increased substantially as the amount of alcohol normally purchased by a household increased, with the top one fifth of households that normally bought the most alcohol increasing their purchases more than 17 times than the bottom one fifth of households that bought the least alcohol. That the heaviest buyers of alcohol increased their purchases the most, with some independent impact of socio-economic disadvantage, might explain why reported alcohol problems and recent alcohol-related death rates might have increased. A conclusion of this is that alcohol policy to reduce high consumption of alcohol, and the availability of help and treatment to reduce alcohol consumption become more important during extraordinary times, such as COVID lockdowns.

## Introduction

During 2020, COVID-19 mitigation measures were put in place in Great Britain (England, Scotland and Wales) in an attempt to control the spread of the virus and limit its impact on public services. The level and geographic scope of restrictions varied considerably over the year, from the initial national ‘lockdown’ implemented at the end of March, with some relaxation during May and June, to ongoing restrictions for the rest of the year that differed across and within England, Scotland and Wales, in terms of non-essential travel, social gatherings, and openings and controls on a large range of retail, hospitality and leisure outlets [1–3], Table 1.

**Table 1:** COVID-19 lockdown regulations affecting the on-trade in England, Scotland, and Wales during 2020.

Date	England	Scotland	Wales
**26 March**	National lockdown measures implemented. All non-essential high street businesses were closed. People were ordered to stay home, leaving for essential purposes only.		
**4 July**	Lockdown regulations relaxed, pubs and restaurants permitted to reopen.		
**13 July**			Pubs and restaurants permitted to reopen.
**15 July**		Pubs and restaurants permitted to reopen.	
**3 August**	Month-long "Eat Out to Help Out" scheme begins, with government subsidized meals at indoor venues.		
**24 September**	New regulations prohibit pubs and restaurants from operating between 22:00 and 05:00.		New regulations prohibit pubs and restaurants from operating between 22:00 and 05:00.
**9 October**		Pubs and restaurants in some areas closed. Licensed premises in other areas opened for outdoor service only. Cafes could open but not sell alcohol.	
**14 October**	Regional 3 Tier system introduced^1^. In Tiers 1 & 2 hospitality businesses were required to close at 22.00. In Tier 3, pubs and restaurants could also only serve alcohol with a “substantial meal”.		
**23 October**			"Short, sharp" national lockdown implemented, pubs, and restaurants closed.
**2 November**		5 Level Protection Measures (0–4) introduced^2^. Levels 0, 1 & 2, pubs and restaurants could open with time restrictions; Level 3 alcohol on-sales permitted; Level 4 pubs and restaurants were closed.	
**5 November**	National restrictions reintroduced, pubs and restaurants closed although food and drink sales for consumption at home permitted.		
**9 November**			National lockdown ended, pubs and restaurants reopen.
**2 December**	3 Tier system reintroduced		
**19 December**	New Tier 4 introduced^3^ requiring pubs and restaurants to close, although food and drink sales for consumption at home permitted.		
**25 December**	Restrictions relaxed for Christmas Day to allow people to mix indoors and travel more freely.		
**26 December**	Majority of UK moves into Tier/Level 4 restrictions.		

^1^Tier regulations in England: Tier 1 = Local COVID alert level medium; Tier 2 = Local COVID alert level high; Tier 3 = Local COVID alert level very high. https://tinyurl.com/3jr2765v.

^2^Protection measure levels in Scotland: Level 0 = lowest; Level 4 = highest, https://tinyurl.com/2f8kku56.

^3^Tier 4 = “Stay at home” highest alert, https://tinyurl.com/rdafk5bk.

With respect to alcohol, on-trade outlets (pubs, bars and restaurants) were fully closed from 21^st^ March to 4^th^ July (England), 13^th^ July (Wales) and 15^th^ July (Scotland). From July onwards, restrictions varied according to local authority and devolved status (England, Scotland or Wales), affecting the size and type of social gatherings, and whether, where and when on-trade alcohol sales could take place, i.e., outside or inside the premises, at the bar or only at tables, and in terms of licencing hours. Some repeated shorter term full closures of on-trade premises were introduced during October and November, and at the end of December [1–3].

Many studies have suggested that COVID-19 confinements and other mitigation measures have led to reporting of increased mental health problems, including anxiety and depression [4, 5]. For alcohol consumption, however, a mixed picture has emerged, at least in Great Britain. On the one hand, population survey data has suggested that there have been increases in alcohol consumption during this period [6–8], and mortality registration data has shown an increase in alcohol-related deaths in England and Wales [9]. On the other hand, analyses of off-trade purchase and sales data [10, 11] found no increase in purchases or sales, when taking into account foregone purchases due to pub closures.

One explanation of these differences could be that, whereas overall levels of alcohol sales and consumption have not increased, the distribution of changes within the population during lockdown has varied. During complete or partial confinement restrictions, people at higher risk of alcohol related harm, such as those already drinking heavily, or in lower socioeconomic groups, may have been over-represented in surveys as opposed to lighter drinkers [12].

Using British household data for the six years 2015 to 2020, we study the impact of COVID-19 on alcohol purchases and consider the extent to which any changes varied over time and by household characteristics, such as age of main shopper, household income, social grade, area of residential deprivation and how much alcohol households normally purchased. Specifically, we ask if households that normally bought more alcohol, and households with indices of deprivation increased their purchases disproportionally.

## Methods

### Study design

We undertook time-controlled, interrupted time series regression analyses of the impact of COVID-19 lockdown introduced on 23^rd^ March 2020 on household purchases of alcohol during the remainder of 2020, using purchases averaged over the period 1^st^ January 2015 to 31^st^ December 2019 as controls.

### Data source

Our data source is Kantar Worldpanel’s (KWP) household shopping panel, which we have previously described [10]. KWP comprises approximately 30,000 British households at any one time, recruited via stratified sampling, with targets set for region, household size, age of main shopper, and occupational group. The same households provide longitudinal data over time. Although there is movement of households, with some households leaving and others joining the panel, in general, the panel remains representative of households in Great Britain as a whole. Households provide demographic information when joining the panel (age of the main shopper, number of adults in the household, income, and social grade based on occupation), followed by annual updates. Households record all off-trade purchases from all store types, including Internet shopping, brought back into the home using barcode scanners. To be included in KWP’s final datasets, households must meet quality control criteria (meeting thresholds for data recording and purchasing volume or spend (based on household size) every four weeks), with some 90–95% of households included [13]. Panellists also upload digital images of checkout receipts, which KWP uses to verify the accuracy of scanner data.

We obtained raw KWP data on take-home purchasing of alcohol products in Great Britain (England, Scotland and Wales) for the six full years covering 2015–2020, with the truncated postcode (up to first four characters, two letters and two numbers) of each household. The data we obtained had no missing values, with the exception of household income, for which just over one in six households (15.7%) did not provide household income data, with this proportion roughly constant over the six years. We imputed the missing income data using monotonic multiple imputation [14]. Alcohol purchases are recorded daily. For each individual purchase, the data includes the type and volume of the purchase using 19 drink categories, the brand, and the alcohol by volume (ABV). The volume purchased was combined with ABV to calculate grams of alcohol purchased.

We grouped households into: (i) three groups of the age of the main shopper (18–44; 45–64; 65+); (ii) five social grades (AB, C1, C2, D, E), based on the National Readership Survey categories, with AB including higher and intermediate managerial, administrative and professional occupations, and E including those with state pensions only, casual workers and unemployed with state benefits only [15]; (iii) five similar sized household income groups (£0–7.5k; >£7.5–12.5k; >£12.5–15.5k; >£17.5 to 25k; >£25k per adult per household per year); (iv) five similar sized groups of the number of grams of all alcohol regularly purchased (>0–7; >7–14; >14–28; >28–70; and >70 grams of alcohol purchased per adult per household per seven days averaged over total number of days between first and last recorded day of an alcohol purchase); and, (v) quintiles of deprivation ranging from 1 (most deprived) to 5 (least deprived) based on multiple indices of deprivation aggregated at truncated postcode level for each of England [16], Scotland [17] and Wales [18].

We prepared data for each day of each calendar year (2015 to 2020) for the interrupted time series analyses by, first, for any day that a household bought alcohol, summing the amount of alcohol purchased in grams, divided by the number of adults in the household. Then, for each day of each year (2015 to 2020), we calculated the sum of purchases across all households.

We then generated a new series of dependent variables for each day of the year, representing the differences between 2020 and the average of 2015–2019 for the sum of purchases across all households. To assess the impact of COVID-19 lockdowns, we undertook interrupted time series analyses, with the one event, the introduction of confinement on 23^rd^ March 2020.

### Statistical analyses

We present descriptive socio-demographic statistics of the households, and estimate Mantel-Haenszel common odds ratios (with 95% confidence intervals) for households with the age of the main shopper 65 years or more compared to households with younger ages and for households in the north of England (North West, North East and Yorkshire and Humber regions) compared with the rest of Great Britain for a range of socio-demographic characteristics.

For the interrupted time series analysis, the dependent variable was 2020 minus the average of 2015–2019 for the sum of purchases in grams of alcohol per adult per household per day across all households for each day of the year, converted to a per cent scale, where 100% is the mean of the sum of purchases per day for the average of the days of 2015–2019 up to 22^nd^ March, covering the days of the year before confinement. For the newly created dependent variable, we examined the distribution visually and with Q-Q plots and found normal distribution. Based on the Durbin-Watson statistic (1.965), there was no evidence of autocorrelation for the series over time. We examined the immediate and permanent level changes due to the event—the introduction of confinement—at day 83 of the year. The event variable was entered as a dummy variable, coded with 0 for each day before the event and with 1 for each day from the event forwards. The regression equation is:

Difference in purchase (2020 minus average of 2015–2019 on the percent scale as described in the preceding paragraph) = intercept + time + event + error, where time is day of the calendar year, and the event is the dummy-coded variable for the introduction of lockdown.

To consider if changes in purchases following confinement differed by household characteristics, we undertook Poisson regression to model the changes in purchases (2020 minus average of 2015–2019) separately by the levels of each of the household groupings of amount of alcohol purchased, age, income, social grade, deprivation, and region of Great Britain as categorical variables, with one category for each grouping assigned as reference category. For all models, we examined the Pearson Chi-Square as part of the goodness of fit statistics, and found no evidence of over dispersion (i.e., values were near to, but less than 1.0). We report the exponential value of the coefficient (and 95% confidence intervals), which is an incident rate ratio (IRR). For example, if the South-West region of England is assigned as reference category, and the IRR for the North-East of England is 1.37, this means that the increase in alcohol purchases in the North-East of England following confinement is 1.37 times higher than the increase in the South-West of England.

#### Accounting for foregone on-trade purchases

Over the time period 2015 to 2019, approximately 29% of total alcohol was purchased on-trade (from pubs, bars, restaurants etc.) in Great Britain (England, Scotland and Wales) [19]. For the three months or so following the introduction of confinement, sales of on-trade alcohol were not possible, and, thereafter, were partially or, for limited times, fully restricted [1–3]. To account for the potential foregone purchases of on-trade alcohol, we redid the analyses by simply adding the potential foregone purchases to the daily household purchases for the average of each day of 2015–2019 since the 26^th^ March of each year.

All analyses were performed with SPSSv26 [20].

## Results and discussion

Data for just over five million separate alcohol purchases were provided by 79,417 households over the six years from 2015 to 2020. Of the households, 70,903 provided purchase data for 2015 to 2019, and 29,890 for 2020. The distribution of socio-demographic factors of the households for the two time periods is illustrated in Table 2.

**Table 2:** Distributions of socio-demographic characteristics of households for the two time periods, 2015 to 2019 and 2020.

		Period 2015 to 2019	Period 2020
		N 70,903 households	N 29,890 households
**Purchase group (grams)**	>0–7	22.9%	20.0%
	>7–14	16.9%	16.0%
	>14–28	18.4%	18.7%
	>28–70	20.6%	23.2%
	>70	21.2%	22.1%
	**Total**	**100.0%**	**100.0%**
**Age group (years)**	18–44	41.0%	34.0%
	45–64	41.1%	44.0%
	65+	17.9%	22.1%
	**Total**	**100.0%**	**100.0%**
**Social Grade group**	AB	20.7%	21.2%
	C1	39.4%	41.2%
	C2	18.1%	18.0%
	D	13.9%	12.8%
	E	8.0%	6.8%
	**Total**	**100.0%**	**100.0%**
**Household income group**	£0–7.5k	21.7%	17.0%
	>£7.5–12.5k	20.3%	20.3%
	>£12.5–15.5k	22.2%	22.4%
	>£17.5 to 25k	16.7%	18.9%
	>£25k	19.0%	21.5%
	**Total**	**100.0%**	**100.0%**
**Area-based deprivation group**	1.00 (most deprived	4.9%	4.7%
	2.00	24.4%	24.2%
	3.00	36.3%	36.2%
	4.00	27.0%	27.2%
	5.00 (least deprived)	7.4%	7.7%
	**Total**	**100.0%**	**100.0%**
**Region of Great Britain**	North East	4.9%	4.8%
	North West	11.0%	11.1%
	Yorkshire and The Humber	10.1%	10.0%
	East Midlands	9.0%	9.0%
	West Midlands	8.8%	8.8%
	Eastern	10.7%	10.9%
	London	7.2%	7.2%
	South East	15.0%	14.9%
	South West	9.7%	9.6%
	Scotland	8.4%	8.5%
	Wales	5.1%	5.1%
	**Total**	**100.0%**	**100.0%**

Households with older residents (age of main shopper) were more likely to be heavier purchasers of alcohol, be classified as social grade E and have a lower income, but not be situated in a more deprived residential area, than households with younger residents, Table 3. Households in the north of England were slightly more likely to be heavier purchasers of alcohol, to have older residents (age of main shopper), be classified as social grade E, and to have a lower income, and considerably more likely to be situated in a more deprived residential area.

**Table 3:** Differences in socio-demographic factors by age of main shopper and geographic location of household.

	Proportions (%) of age group 18–64 years (age of main household shopper)	Proportions (%) of age group 65+ years (age of main household shopper)	Odds ratio (95% CI) for age group 65+ years compared to age group 18–64 years
**Purchase group > 70 grams per adult per seven days**	20.3%	26.6%	1.42 (1.37 to 1.47)
**With social grade E**	6.0%	14.4%	2.61 (2.49 to 2.75)
**With household income £0–7.5k per adult**	18.1%	30.7%	2.01 (1.93 to 2.09)
**Living in most deprived area**	5.0%	4.2%	0.84 (0.77 to 0.90)
	**Proportions (%) of rest of Great Britain**	**Proportions (%) of north of England (North East, North West and Yorkshire and The Humber)**	**Odds ratio (95% CI) for north of England compared to rest of Great Britain**
**Purchase group > 70 grams per adult per seven days**	21.1%	22.7%	1.10 (1.06 to 1.14)
**With age of main household shopper 65+ years**	18.8%	20.2%	1.10 (1.06 to 1.14)
**With social grade E**	7.5%	8.1%	1.09 (1.04 to 1.15)
**With household income £0–7.5k per adult**	19.8%	21.7%	1.13 (1.09 to 1.17)
**Living in most deprived area**	2.9%	10.4%	3.90 (3.68 to 4.14)

**Fig 1:**
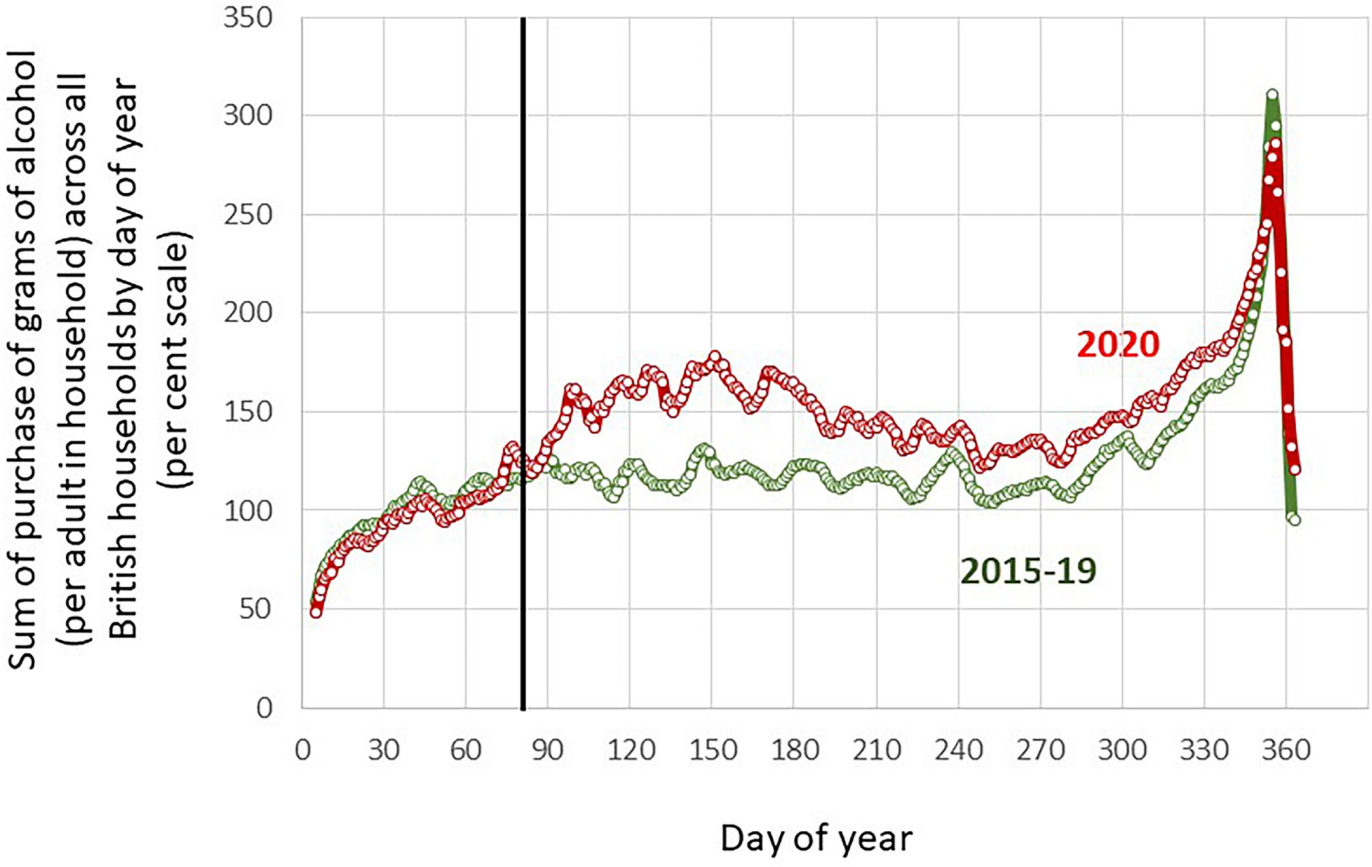
Plots of purchases of grams of alcohol by day of year for 2015–2019 (averaged) and 2020. Purchases are the sum of purchases in grams of alcohol per adult per household per day across all households for each day of the year, converted to a per cent scale, where 100% is the mean of the sum of purchases per day for the average of the days of 2015–2019 up to 22^nd^ March, covering the days of the year before confinement.

Plots of purchases by calendar day of year for the average of 2015–2019 and for 2020 demonstrates parallel trends between the two time periods, confirming the appropriateness of 2015–2019 as a control period, Fig 1.

### Impact of COVID-19 confinement

Confinement was associated with an increase in purchases of grams of alcohol per adult per household of:

■ 29.2% (95% CI = 25.8% to 32.5%) over all days post the introduction of confinement from 23^rd^ March to 31^st^ December;
■ 37.5% (95%CI = 33.9 to 41.2) for the time-period 23^rd^ March to 15^th^ July, coinciding with lockdown; and,
■ 24.6% (95%CI = 21.6 to 27.7) for the time period 16^th^ July to 31^st^ December, when lockdowns were eased.

From 16^th^ July to 31^st^ December, increased purchases remained relatively stable over time, with a coefficient of -0.025% per day over time (95% CI = -0.061 to 0.011).

To account for the potential foregone purchases of on-trade alcohol due to on-trade closures or sales restrictions, we redid the analyses by simply adding the potential foregone purchases to the daily average household purchases for each day of 2015–2019 since the 23^rd^ March. In this hypothetical scenario, there was no change in alcohol purchases (2020 minus average of 2015–2019) following confinement between 23^rd^ March and 15^th^ July (coefficient = -0.90, 95%CI = -5.58 to 3.77), but decreased purchases for the rest of the year (coefficient = 29.62%, 95%CI = 23.88 to 35.35).

### Increase in purchases by socio-demographic characteristics of households

Table 4 lists the incidence rate ratio for increased purchases of grams of alcohol by household characteristics. The greater the amount of alcohol normally purchased by a household, the greater the ratio of increase of alcohol purchases following confinement. The highest purchasing households increased their purchases of alcohol following confinement more than 17 times compared to the lowest purchasing households. Households where the age of the main shopper was 65 years or older increased their purchases more than households where the main shopper was aged younger. Households from social grade E (which includes those with state pensions only, casual workers and unemployed with state benefits only) increased their purchases more than households in grade AB (which includes those with higher and intermediate managerial, administrative and professional occupations); however, those in grade D, which includes manual workers, had a lower increase in purchases than households in grades AB. Higher income households increased their purchases more than lower income households. For residential deprivation, those in the most deprived group increased their purchases more than those in the least deprived group. In general, households in the north of England (except for the north-west) increased their purchases by more than households in the south of England. Households in Scotland and Wales increased their purchase by a much smaller amount compared to the regions of England.

**Table 4:** Incidence rate ratio of increased purchases of alcohol (95% confidence intervals) by household characteristics.

	Incidence rate ratio by household characteristics
**Purchase group**	
>70	17.25 (14.75 to 20.19)
>28–70	11.38 (9.70 to 13.34)
>14–28	5.96 (5.06 to 7.03)
>7–14	4.80 (4.06 to 5.68)
0–7 (reference category)	1.00 (0. to 0.)
**Age group**	
25–44	0.79 (0.75 to 0.84)
45–64	0.67 (0.63 to 0.71)
65+ (reference category)	1.00 (0. to 0.)
**Social grade**	
E	1.59 (1.51 to 1.68)
D	0.79 (0.74 to 0.85)
C2	1.06 (1.00 to 1.12)
C1	1.07 (1.01 to 1.13)
AB (reference category)	1.00 (0. to 0.)
**Household income**	
£0–7.5k	0.92 (0.87 to 0.98)
>£7.5–12.5k	0.63 (0.59 to 0.67)
>£12.5–15.5k	0.73 (0.69 to 0.78)
>£17.5 to 25k	0.64 (0.60 to 0.68)
>£25k (reference category)	1.00 (0. to 0.)
**Area-based Deprivation group**	
1 (most deprived)	1.13 (1.06 to 1.19)
2	0.99 (0.94 to 1.05)
3	0.81 (0.76 to 0.86)
4	1.05 (0.99 to 1.11)
5 (least deprived) (reference category)	1.00 (0. to 0.)
**Region**	
Scotland	0.53 (0.49 to 0.57)
Wales	0.48 (0.44 to 0.51)
North East	1.37 (1.30 to 1.45)
North West	0.79 (0.74 to 0.84)
Yorkshire and The Humber	1.72 (1.63 to 1.81)
East Midlands	1.09 (1.03 to 1.15)
West Midlands	1.01 (0.95 to 1.07)
Eastern	1.06 (1.00 to 1.12)
London	0.92 (0.87 to 0.98)
South East	0.90 (0.85 to 0.96)
South West (reference category)	1.00 (0. to 0.)

## Discussion

We show that the introduction of COVID-19 confinement in Great Britain towards the end of March 2020 was associated with households buying 29% more off-trade alcohol (expressed as grams of alcohol) for the rest of the year than would have been expected based on average purchases throughout 2015–2019. The increase was greater (38%) during the times of full pub closure (end of March to early to mid-July 2020) than for the rest of the year (25%).

However, when taking into account that households could not buy on-trade alcohol from the end of March to early to mid-July 2020 (as outlets were closed) and that there were continuing restrictions for the rest of the year in purchasing on-trade alcohol, a different picture emerges. In this hypothetical scenario, during the times that pubs were closed, there was no change in the overall amount of alcohol that households purchased (combining off-trade and on-trade inferred from previous years’ purchasing profiles), and a decrease in purchases thereafter.

Increases in purchases were predominantly driven by households that were usually the highest purchasers of alcohol. The top one fifth of purchasing households (by how much they normally purchased) increased their purchases 17 times more than the bottom one fifth of purchasing households. There was some evidence to suggest that the most disadvantaged households increased their purchases more than the least disadvantaged households, based on social grade and deprivation index, and, to some extent, on household income. In general, households in the north of England increased their purchases more than households in the south of England and the rest of Great Britain, probably because households in the north of England tended to be more disadvantaged and to some extent heavier purchasers of alcohol in general than households elsewhere.

That households with older residents (as measured by age of main shopper) increased their purchases more than households with younger residents may be partly explained by the fact that such households tended to be heavier purchasers of alcohol in general, and to be more disadvantaged than households with younger residents.

There is a paradox when comparing our results with other analyses. On the one hand, our results are consistent with customs and excise tax data [21, 22] and other sales data that showed no overall increase in alcohol purchases following COVID-19 confinement [11]. On the other hand, our results do not confirm the self-reported increases in alcohol consumption due to lockdown found in surveys [8, 23–25]. However, that heavy buying households increased their purchases and that, to some extent, the most disadvantaged households increased their purchases more than the least disadvantaged households, might explain the increases in alcohol consumption reported in some surveys and the increase in alcohol-related deaths reported in England & Wales [9].

Data from the behavioural risk factor surveys of Public Health England [8] show changes in patterns of drinking following confinement, with more non-drinkers, less lighter drinkers and more heavier drinkers following lockdown. In the surveys, the changes occurred across all age groups, but increases in the proportion of heavy drinkers were less in the younger and older age groups, and more in the middle aged groups (35–64 years), dissimilar to our results where increases in purchases were greater in those aged 65 plus years than the younger age groups. In the surveys, there was a steep social grade gradient with greater increases in the proportion of heavy drinkers moving from social grade group E to group A, whereas we found a greater increase in purchases in social grade group E, compare with AB, but a much lower increases in purchases in social grade group D compared with group AB. In the surveys, increases in the proportion of heavy drinkers occurred across all regions of England, with the increases being higher in the north, rather than in the south of England, similar to our findings. Increases in purchases were much lower in Scotland and Wales than in England.

Data from the Office for National Statistics for England and Wales finds an increase in wholly attributable alcohol-related deaths during 2020 compared to previous years [9]. Compared with 2019, whilst deaths were 8.5% higher during the first quarter of 2020 (prior to confinement), deaths were 17.4% higher during the second quarter, 21.9% higher during the third quarter, and 28.3% during the fourth quarter. There were variations by region, with the increase in deaths being higher in the north as opposed to the south of England, mirroring the changes in purchases that we found. Further, deaths in Wales decreased during the last three quarters of 2020. In England, there was no huge difference in changes in deaths by deprivation quintile. For men, during 2020, the increases in the most deprived quintile were 4.2 times greater than in the least deprived quintile, compared to a 3.8-fold difference in 2019. For women, the respective differences were 3.0 in 2020 and 3.2 in 2019.

Putting all of this together would suggest that heavier and more socially disadvantaged drinkers were disproportionately affected by drinking more alcohol following COVID-19 lockdown than lighter and less socially disadvantaged drinkers. This would imply that during such times, there is a need to strengthen implementation of alcohol policy measures to reduce the harm done by alcohol, such as those put forward by the WHO SAFER initiative [26], as well as strengthen structural policies that help to improve all people’s socioeconomic prospects, aligned with approaches that address the social determinants of health [27, 28].

Our analyses have several important strengths. We obtained product bar code data from a large number of households, with a large number of daily data points before and after the analyzed event (the introduction of COVID-19 lockdown). We undertook controlled interrupted time series analyses, using purchases over the years 2015 to 2019 as time controls for 2020, subtracting the differences between the respective time periods for our analyses. The use of time controls helps to control for any confounding events that would affect both time periods, such as limitations related to data collection.

As we have noted in previous publications, analyses of such household purchase data have some limitations [10, 29–32]. A key limitation of our study is that, except for the purchases during the period of COVID-19 confinement (between 23^rd^ March and 4^th^ July 2020, when on-licensed premises were closed, with, in principle, all legal alcohol purchases captured), we only measure off-trade alcohol purchases and not on-trade purchases. The data also has limitations, with heavy drinkers tending to be under-represented in household panel data [33, 34], with alcohol purchases tending to be under-reported in these datasets [35, 36]. It may also be the case that such under-recording of alcohol is higher among households purchasing the highest levels of alcohol. Additionally, we are only able to assess changes in off-trade alcohol purchases as opposed to actual levels of alcohol consumption for these time periods. Adults in a household may not have an equal share of the alcohol purchased, and not all adults in a household may be drinkers of alcohol.

## Conclusions

There is a long-standing and well characterized alcohol harm paradox where alcohol-related harms are disproportionally experienced by people living in lower socio-economic status groups despite reported heavy drinking across all socio-economic status groups. Here we see a new paradox in which British data suggest no increases in overall sales and purchase of alcohol following COVID-19 lockdowns, yet survey and mortality data suggesting otherwise is due to differential changes by population sub-groups. The top one fifth of purchasing households (by how much they normally purchased) increased their purchases 17 times more than the bottom one fifth of purchasing households. The most disadvantaged households increased their purchases more than the least disadvantaged. Further, households in the north of England increased their purchases more than households in the south of England, mirroring changes in alcohol-specific death rates during 2020 compared with previous years. This suggests that alcohol policy to reduce high consumption of alcohol becomes more important during extraordinary times, such as COVID lockdowns. That the increase in purchases was much less pronounced in Scotland and Wales, compared with England, could be attributed to the minimum unit price of alcohol introduced in both jurisdictions, such a policy shown to reduce alcohol purchases, particularly amongst the heaviest purchasing households during times of lockdown [30].
